# Vascular Endothelial Growth Factor Receptor-1 Expression in Breast Cancer and Its Correlation to Vascular Endothelial Growth Factor A

**DOI:** 10.1155/2013/746749

**Published:** 2013-12-12

**Authors:** Nahida Srabovic, Zlata Mujagic, Jasminka Mujanovic-Mustedanagic, Adaleta Softic, Zdeno Muminovic, Adi Rifatbegovic, Lejla Begic

**Affiliations:** ^1^Department of Biochemistry, University of Tuzla, 75 000 Tuzla, Bosnia And Herzegovina; ^2^Policlinic of Laboratory Diagnostics, Department of Pathology, University Clinical Centre of Tuzla, 75 000 Tuzla, Bosnia And Herzegovina; ^3^Policlinic of Surgery, University Clinical Centre Tuzla, 75 000 Tuzla, Bosnia And Herzegovina

## Abstract

VEGF-A is the most potent angiogenic factor in tumour angiogenesis. Its effects are mediated via two receptors VEGFR-1 and VEGFR-2. Primary aim of our study was to examine the expression of VEGFR-1 in breast cancer and its correlation to VEGF expression, lymph node status, tumour size, histological grade, and hormone receptor status. To examine the VEGFR-1 and VEGF expressions in tumour and surrounding tissue of 51 breast cancer patients, and in healthy breast tissue of 30 benign breast diseases patients, we used three-step immunohistochemical staining. VEGFR-1 and VEGF expressions were significantly increased in breast cancer tumour in relation to surrounding tissue (*P* < 0.01), and the VEGF expression was significantly increased in lymph node positive breast cancer patients (*P* < 0.01). VEGFR-1 and VEGF expressions were significantly higher in breast cancer tumour compared with healthy breast tissue (*P* < 0.01). Significant correlation between VEGF and VEGFR-1 expressions was found (*P* < 0.05). No significant correlations between VEGF and VEGFR-1 expressions and tumour size, histological grade, and hormone receptor status were found. Increased expression of VEGFR-1 and VEGF in breast cancer tumour and significant correlation between these proteins suggest the possible role of VEGF/VEGFR-1 signalization in breast cancer development, although VEGFR-1 potential prognostic value was not confirmed.

## 1. Introduction

Breast cancer is the most common cancer and the leading cause of cancer death among women worldwide [1]. Like many other solid cancers, it requires an independent blood supply to enlarge and develop metastases [2, 3]. This process, called tumour angiogenesis, is mediated primarily by vascular endothelial growth factor A (VEGF-A) [4]. A lot of studies have suggested that VEGF expression in tumour tissue is significantly correlated with microvessel density (MVD) and poor prognosis in human cancers including breast cancer [5, 6].

VEGF activates tyrosine kinase receptors, VEGFR-1 (also referred to as FLT1) and VEGFR-2 (or KDR) located in the endothelium, which leads to stimulation of endothelial migration, proliferation, permeability, and survival [7, 8]. Monocyte/macrophages, haematopoietic stem cells, and some tumour cells also express VEGFR-1 [9–11]. Most of the previously mentioned results of VEGF activity are consequences of VEGFR-2 activation [5]. VEGFR-1 has a weak tyrosine kinase activity but higher binding affinity for VEGF than VEGFR-2 [12]. To date, the role of VEGFR-1 in angiogenic signal delivery for VEGF in tumour angiogenesis is poorly examined and still not entirely clear. Although mice that lack a functional VEGFR-1 develop normally indicating that the role of the VEGFR-1 in physiological angiogenesis is not required [13], role for this receptor during tumour angiogenesis has been recently suggested [14–18]. On the other hand, VEGF induces vascular permeability which is necessary for physiological processes such as wound healing, but it might also promote formatting oedema and ascites, facilitating the distant spread of metastases in cancer patients [19]. VEGF activation of VEGFR-2 leads to angiogenic effects without signs of oedema [20], which implicates that signalling through VEGFR-2 alone does not induce permeability, rather cross communications between VEGFR-1 and VEGFR-2 [21].

Although few studies have suggested that expression of VEGFR-1 in human breast cancer tumour tissue are increased and correlated with prognostic factors [15, 16], precise mechanism, biological effect, and therapeutic impact of the VEGF/VEGFR-1 signalling are mostly unknown.

Primary aim of our study was to investigate the expression of the VEGFR-1 and VEGF in primary breast cancer tumour and their expression in surrounding tissue and in the healthy breast tissue of the patients with benign breast diseases. Further, we assessed whether VEGF/VEGFR-1 signalling is a prognostic factor by testing our findings against established prognostic and predictive factors such as nodal status, tumour size, histological grade, and hormone receptor status of examined tumours. The results from this study may contribute to understanding VEGF/VEGFR-1 signalling in breast cancer and also may contribute to reassessing anti-VEGF/VEGFR therapy for breast cancer.

## 2. Material and Methods

### 2.1. Patients

Total of 51 invasive breast cancer patients, subjected to the breast surgery at the Department of Surgery, University Clinical Centre Tuzla, without adjuvant chemotherapy and other major illnesses, were included in this study (21 without metastases in axillary's lymph nodes and 29 with metastases in axillary's lymph nodes). A number of 30 patients with benign breast diseases, who were also subjected to the appropriate breast surgery, were used as control group.

The study was approved by Ethic committee of the University Clinical Centre Tuzla.

### 2.2. Tissue Samples

Tumour and breast tissue samples that have been obtained during surgery were sent to the Department of pathology, University Clinical Centre Tuzla, to carry out the regular pathological examination and pathohistological diagnosis. Tumour tissue samples and the samples of surrounding tissue from patients with invasive breast cancer were used, as well as normal breast tissue samples from patients with benign breast diseases. Samples have been collected during twelve months in 2011.

Pathohistological examination was performed with hematoxylin-eosin staining. Tumours were classified according to the criteria of the World Health Organization [22]. Histological grade was determined in accordance to the modified Scarff-Bloom-Richardson grading system. Tumour size was graded in three categories: I (tumour size was 0,1–2 cm), II (tumour size was 2–5 cm), and III (tumour size was >5 cm).

### 2.3. Immunohistochemistry

Three-step immunohistochemical procedure for estrogens receptor (ER), progesterone receptor (PR), vascular endothelial growth factor A (VEGF-A), and VEGF receptor, 1 (VEGFR-1) was performed. Deparaffinization and rehydration of 4 *μ*m thick formalin-fixed paraffin embedded sections were performed in xylene and ethanol solutions (decreasing concentration 96–70%). To block endogenous peroxidase, sections were incubated in H_2_O_2_ solution (1,5% H_2_O_2_ in methanol) for 15 minutes. Antigen retrieval was performed in procedure with the buffer (pH = 9.0, TRIS 20 mmol/L, EDTA 0.05 mmol/L, 0.05% Tween 20) in a microwave oven by heating the slides for 15 minutes. After rinsing with the PBS buffer, normal goat serum (for ER and PR) (DAKO, Denmark) and Protein block (for VEGF and VEGFR-1) (Mouse and rabbit specific HRP plus detection IHC kit, Abcam,plc, Cambridge, UK), respectively, were applied for 15 minutes at room temperature to block nonspecific antibody binding. After that, sections were incubated for an hour with primary antibody at 37°C. After rinsing with the PBS buffer, the secondary antibody (for ER and PR) and biotinylated goat-polyvalent plus (for VEGF and VEGFR-1) (Mouse and rabbit specific HRP plus detection IHC kit, Abcam,plc, Cambridge, UK), respectively, were applied for 30 minutes at room temperature. After rinsing with the PBS buffer streptavidin-HRP (for ER and PR) (DAKO, Denmark) and streptavidin peroxidase plus (for VEGF and VEGFR-1) (Mouse and rabbit specific HRP plus detection IHC kit, Abcam,plc, Cambridge, UK), respectively, were applied for 30 minutes at room temperature. As chromogen we used diaminobenzidine (Fluka Chemie, Switzerland). For counterstaining, we used hematoxylin (Fluka Chemie GmbH, Buchs; Switzerland). Slide preservation was performed in Canada balsam (turpentine). Dilutions of primary and secondary antibodies were as follows:

- a mouse antihuman monoclonal antibody against ER, clone NCL-ER-6F11 (Novocastra, UK) in the PBS/BSA buffer, pH = 7.2;
- a mouse antihuman monoclonal antibody against PR, clone NCL-PGR-312 (Novocastra, UK) dilution 1 : 150 in the PBS/BSA buffer, pH = 7.2;
- a mouse antihuman monoclonal antibody against VEGF that detects the 121, 165, and 189 VEGF isoforms in routinely fixed specimens, clone VG-1 (Abcam,plc, Cambridge, UK) dilution 1 : 200 in the PBS/BSA with NaN3 buffer;
- a rabbit antihuman monoclonal antibody against VEGFR-1 that recognises VEGFR-1 and its splice isoform sFlt1 in routinely fixed specimens, clone Y103 (Abcam,plc, Cambridge, UK) dilution 1 : 220 in the PBS/BSA with NaN3 buffer;
- a goat antimouse polyclonal antibody biotin conjugated (DAKO, Denmark) dilution 1 : 200 in the PBS/BSA buffer, pH = 7.2.

Tissue sections of breast cancer previously fortified index immunoreactivity were used as positive controls for ER and PR, and endothelial cells of breast tissue were used as positive control for VEGF and VEGFR-1. Sections from the same paraffin blocks also used as negative control but during immunohistochemical staining specific primary antibody were replaced with normal species (the same species as primary antibody).

### 2.4. Immunohistochemical Staining Evaluation

The final immunohistochemical staining evaluation for ER and PR was performed by pathologists using light microscopic analyzing (light microscope, Olympus Medical Systems Corp., Japan). Evaluation was performed by using Quick Score immunoreactivity score [23, 24] for ER- and PR-immunoreactivity.

Due to some universally accepted criteria, the final immunohistochemical staining evaluation scoring for VEGF and VEGFR-1 expression was developed by authors. The expression of VEGF and VEGFR-1 was scored as 0 for no immunoreactivity staining, +1 for poorly staining, +2 for moderately staining, and +3 for strongly staining.

### 2.5. Statistical Analysis

The results were evaluated by Wilcoxon test for dependent sample, Mann-Whitney *U* test for independent sample, and with Spearman correlation. For all performed tests, *P* < 0.05 was considered as statistically significant. For statistical analyses, we used SPSS 17.0 software (SPSS Inc., USA).

## 3. Results

From a total of 51 breast cancer patients included in this study, 29 of them (56.86%) had positive lymph nodes. None of patients with negative lymph nodes had category III tumour size (>5 cm). In 13 patients with negative lymph nodes tumour size was in category II (2–5 cm), and in eight patients with negative lymph nodes tumour size was in category I (0,1–2 cm). In six patients with positive lymph nodes tumour size was in category III, and in 16 lymph node positive patients tumour size was in category II, and in eight of them tumour size was in category I.

None of patients with negative or positive lymph nodes had weakly differentiated tumour (grade III): nine patients with negative lymph nodes had moderately differentiated tumour (grade II) and 11 of them had well-differentiated tumour (grade I). Tumour was moderately differentiated in 14 patients with positive lymph nodes and well differentiated in 12 patients with positive lymph nodes.

In patients with negative lymph nodes, ER were positive in 17 patients and negative in four patients, and PR were positive in 17 patients and negative in four patients. In patients with positive lymph nodes, ER was positive in 20 patients and negative in 10 patients, and PR was positive in 21 patients and negative in nine patients.

**Figure 1:**
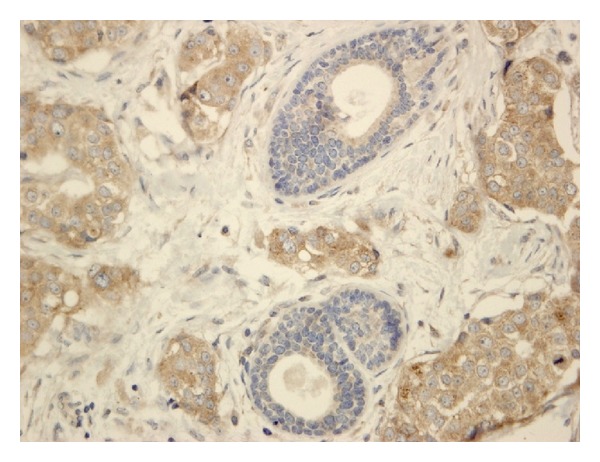
Microphotography of breast cancer tumour with strongly immunohistochemical staining for VEGF (brown staining) (HE; ×40).

**Figure 2:**
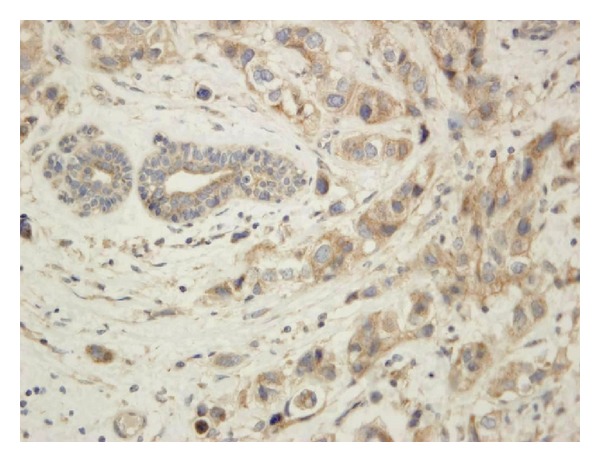
Microphotography of breast cancer tumour with strongly immunohistochemical staining for VEGFR-1 (brown staining) (HE; ×40).

The expression of VEGF and VEGFR-1 was found in breast cancer tumour tissue (Figures 1 and 2) and in surrounding tissue, but it was absent or barely detectable in healthy breast tissue of patients with benign breast disease. The expression of VEGFR-1 and VEGF was distributed mostly in the cytoplasm of the tumour cells; but also their expression was detected in the cytoplasm of the tumour vessel endothelial cells. Both, the expression of VEGF and the expression of the VEGFR-1 were significantly increased in tumour tissue compared to the surrounding tissue in the patients regardless of lymph node status (Tables 1 and 2, resp.). Also the expression of VEGF and the expression of the VEGFR-1 were significantly increased in breast cancer tumour tissue compared with breast tissue of patients with benign breast disease (Tables 1 and 2, resp.). The expression of these proteins was significantly increased in surrounding tissue of the patients with invasive breast cancer compared with healthy breast tissue of patients with benign breast disease (Tables 1 and 2, resp.). Statistically significant correlation between VEGF expression and VEGFR-1 expression in tumour tissue of the patients with invasive breast cancer was found (*P* = 0.017), as well as statistically significant correlation between VEGF expression and VEGFR-1 expression in surrounding tissue of the patients with invasive breast cancer (*P* = 0.003).

The expression of VEGF was significantly increased in tumours of the lymph node positive breast cancer patients in relation to the expression of VEGF in tumours of the lymph node negative breast cancer patients (Table 1). No significant differences in VEGFR-1 expression in tumour tissue considering lymph node status (Table 2) were noticed. No significant correlations of VEGF or VEGFR-1 with tumour size and histological grade were noticed, as well as between VEGF or VEGFR-1 and hormone receptors.

## 4. Discussion

In this study, we found that the expression of VEGFR-1 and VEGF was significantly increased in breast cancer tumour tissue compared with surrounding tissue regardless of lymph node status. We also found that the expression of VEGFR-1 and VEGF was significantly increased in breast cancer tumour tissue compared with healthy breast tissue in the patients with benign breast disease, and its expression was significantly increased in surrounding tissue of breast cancer patients compared with normal breast tissue in patients with benign breast disease. Results obtained in our study are in accordance with the results from prevoius studies [5, 6, 15, 16, 21]. Other studies have showed that the expression of VEGF in tumour is increased and significantly correlated with microvessel density and poor prognosis in human cancers including breast cancer [5, 6]. The role of VEGF in tumour angiogenesis is well known and many of the current anti-angiogenic therapies targeting VEGF for quite some time [4, 25]. VEGF activates VEGFR-2 and VEGFR-1 [7, 8]. VEGFR-2 is the primary VEGF receptor, while the VEGFR-1 is less defined and its role during tumour angiogenesis has been recently suggested [4, 17, 25], although genetic data indicate that signalling trough this receptor is not required for physiological angiogenesis [13]. Expression of VEGFR-1 in tumour cells has been found in human cancers in few other studies [10, 26]. In vitro studies suggested a role for VEGFR-1 signalling in survival of colorectal and pancreatic cancer cell lines during epithelial to mesenchymal transition [27, 28]. Furthermore, antihuman VEGFR-1 mAB treatment increases the survival of mice injected with acute lymphoblastic leukaemia cells [29] and also inhibits tumour growth of VEGFR-1 positive breast cancer and melanoma xenografts [14].

In accordance with all the literature data and results from this study, it is obvious that breast cancer cells and cells in the tumour environment overexpress VEGFR-1. Furthermore, we have found that VEGF significantly correlates with VEGFR-1, and its expression was significantly increased in breast cancer patients with positive lymph nodes. Since VEGF is very potent permeability inducer and vascular permeability is a prerequisite for distant spread of metastases [19], VEGF overexpression in breast cancer patients with positive lymph nodes is understandable and in accordance with the literature data.

On the other hand, we did not found significant differences in VEGFR-1 expression between patients with and without lymph node metastases or significant correlations with pathohistological factors which are not entirely in accordance with the literature considerations. Kiba et al. [29] suggest that signalling through VEGFR-2 alone does not induce permeability. Further, cross communication between VEGFR-1 and 2 might be required. Also, the VEGFR-1-dependent regulated migration of hematopoietic cells has been implicated in the establishment of tumour metastases, as hematopoietic cells home to tumour—specific pre-metastatic sites [30]. Significant correlation between VEGFR-1 and VEGF, that we found, implicates the importance VEGF/VEGFR-1 signalization in breast cancer development, but it is still unclear and needs more research on this subject. VEGF serum level determination when combined with immunohistochemical staining results of VEGF and VEGFR-1 expression may provide more detailed information about this matter and this is important limitation to our study. Besides, tissue expression estimation based on microscopy is probably less objective in relation to some other approaches, for example, the estimation of some housekeeping gene products and probably should be checked in Western blotting. This is also potential limitation of this study.

In conclusion, VEGFR-1 and VEGF are overexpressed in breast cancer tumours and surrounding tissue and mutually correlated. Also, their expression in healthy breast tissue is absent or barely detectable. In accordance to results from our study, VEGFR-1 may not serve as prognostic parameter. Nevertheless, our results may, at least partly, contribute to better understanding, the role of VEGF/VEGFR-1 signalling in the complex breast cancer development and may contribute to reassessing therapeutic approaches to this severe disease.

## Figures and Tables

**Table 1:** The final score VEGF expression in breast cancer patients and in patients with benign breast disease.

	LNT+ (*N*_T_ = 30, *N*_OT_ = 30) *N*, ratio	LNT− (*N*_T_ = 21, *N*_OT_ = 18) *N*, ratio		BBD (*N*_BBD_ = 30) *N*, ratio
*VEGF expression score *				
T				
0	2 (6.67)	2 (9.52)	0	28 (93.33)
+1	18 (60.00)	12 (51.14)		
+2	9 (30.00)	7 (33.33)	+1	2 (6.67)
+3	1 (3.33)	0 (0.00)		
ST				
0	10 (33.33)	3 (16.67)	+2	0 (0.00)
+1	18 (60.00)	15 (83.33)		
+2	2 (6.67)	0 (0.00)	+3	0 (0.00)
+3	0 (0.00)	0 (0.00)		

Differences in VEGF expression between T and ST (Wilcoxon test), T and BT, and T and BT (Mann-Whitney test)	T/ST *z* = −3.77 *P* = 0.0001	T/ST *z* = −2.45 *P* = 0.014	T_LN+_/BT *P* = 0.0001	T_LN-_/BT *P* = 0.0001
	T_LN+_/T_LN-_		ST_LN+_/BT *P* = 0.0001	ST_LN−_/BT *P* = 0.0001
	*P* = 0.002		

LNT+: patients with positive lymph nodes, LNT−: patients with negative lymph nodes, BBD: benign breast disease patients, T: tumour, ST: surrounding tissue, and BT: breast tissue of patients with benign breast disease.

**Table 2:** The final score of VEGFR-1 expression in breast cancer patients and in patients with benign breast disease.

	LNT+ (*N*_T_ = 30, *N*_OT_ = 29) *N*, ratio	LNT− (*N*_T_ = 21, *N*_OT_ = 17) *N*, ratio		BBD (*N*_BBD_ = 30) *N*, ratio
*VEGFR-1 expression score *				
T				
0	1 (3.33)	0 (0.00)	0	25 (83.33)
+1	7 (23.33)	6 (28.57)		
+2	16 (53.33)	12 (57.14)	+1	5 (16.67)
+3	6 (20.00)	3 (14.28)		
ST				
0	2 (6.89)	0 (0.00)	+2	0 (0.00)
+1	17 (58.62)	11 (64.70)		
+2	9 (31.03)	6 (35.30)	+3	0 (0.00)
+3	1 (3.45)	0 (0.00)		

Differences in VEGFR-1 expression between T and ST (Wilcoxon test), T and BT, and T and BT (Mann-Whitney test)	T/ST *z* = −2.961 *P* = 0.003	T/ST *z* = −2.640 *P* = 0.008	T_LN+_/BT *P* = 0.0001	T_LN−_/BT *P* = 0.0001
	T_LN+_/T_LN-_		ST_LN+_/BT *P* = 0.0001	ST_LN−_/BT *P* = 0.0001
	*P* = 0.750		

LNT+: patients with positive lymph nodes, LNT−: patients with negative lymph nodes, BBD: benign breast disease patients, T: tumour, ST: surrounding tissue, and BT: breast tissue of patients with benign breast disease.
